# A marker registration method to improve joint angles computed by constrained inverse kinematics

**DOI:** 10.1371/journal.pone.0252425

**Published:** 2021-05-28

**Authors:** James J. Dunne, Thomas K. Uchida, Thor F. Besier, Scott L. Delp, Ajay Seth

**Affiliations:** 1 Departments of Mechanical Engineering and Bioengineering, Stanford University, Stanford, California, United States of America; 2 School of Sport Science, Exercise and Health, University of Western Australia, Crawley, Western Australia, Australia; 3 Department of Mechanical Engineering, University of Ottawa, Ottawa, Ontario, Canada; 4 Department of Engineering Science, Auckland Bioengineering Institute, University of Auckland, Auckland, New Zealand; 5 Department of Biomechanical Engineering, Delft University of Technology, Delft, The Netherlands; University of Illinois at Urbana-Champaign, UNITED STATES

## Abstract

Accurate computation of joint angles from optical marker data using inverse kinematics methods requires that the locations of markers on a model match the locations of experimental markers on participants. Marker registration is the process of positioning the model markers so that they match the locations of the experimental markers. Markers are typically registered using a graphical user interface (GUI), but this method is subjective and may introduce errors and uncertainty to the calculated joint angles and moments. In this investigation, we use OpenSim to isolate and quantify marker registration–based error from other sources of error by analyzing the gait of a bipedal humanoid robot for which segment geometry, mass properties, and joint angles are known. We then propose a marker registration method that is informed by the orientation of anatomical reference frames derived from surface-mounted optical markers as an alternative to user registration using a GUI. The proposed orientation registration method reduced the average root-mean-square error in both joint angles and joint moments by 67% compared to the user registration method, and eliminated variability among users. Our results show that a systematic method for marker registration that reduces subjective user input can make marker registration more accurate and repeatable.

## Introduction

Computational biomechanical methods provide powerful tools to study human and animal movement. OpenSim is a musculoskeletal modeling and simulation tool that is widely used to assess muscle and joint function [1–4], to predict functional outcomes following interventions [5–7], and to design assistive devices for improving human movement [8–10]. A musculoskeletal simulation must estimate joint angles from experimental data that replicate human and animal movement with sufficient accuracy to achieve the study’s objectives [11].

*Unconstrained inverse kinematics* is a method that uses skin-mounted optical markers to define an anatomical reference frame for each body segment independently, and computes joint angles from the relative orientation of adjacent reference frames [12, 13]. This approach is also referred to as the “direct method” [14–16]; however, we avoid this term to prevent confusion with direct (or forward) kinematics [17], which typically describes the process of computing a model’s end-effector position and orientation in space from known joint angles. Unconstrained inverse kinematics is unsuitable for computing joint angles that will be used for musculoskeletal simulation because this method introduces unrealistic behaviors, such as a model’s body segments changing length and apparent joint dislocations and interpenetrations [18]. These inaccuracies result in unacceptable errors in muscle–tendon lengths and moment arms, making musculoskeletal simulation infeasible.

*Constrained inverse kinematics* is an alternative method that uses a geometric model composed of rigid bodies connected by joints, which prevent body segments from changing length, joints from dislocating, and bones from interpenetrating. Global optimization procedures are used to minimize the weighted least-squares distance between experimental markers and the corresponding markers placed on the computational model [14]. Accurate calculation of joint angles using constrained inverse kinematics requires adjusting the model marker locations to match the locations of the experimental markers, a process called *marker registration*. Several methods have been proposed to improve the accuracy of calculations from constrained inverse kinematics, including optimization of geometric parameters and marker locations [19–22], statistical shape modeling [23], and medical imaging [16, 24, 25]; however, these techniques require specialized software, equipment, and technical skill that may not be readily available. OpenSim provides a graphical user interface (GUI) that allows users to adjust model marker locations to match the placement of experimental markers on the subject, which we refer to as the *user registration* method. However, user registration is subjective and may introduce variability in model marker locations among OpenSim users, leading to uncertainty in calculated joint angles. Ideally, accurate and repeatable results should be obtained regardless of the user’s experience.

The goals of this study were twofold: (i) to assess the variability of user registration on joint angle and moment estimates computed in OpenSim; and (ii) to evaluate a registration method informed only by the location of experimentally placed anatomical markers, which we refer to as the *orientation registration* method. These goals were achieved by comparing marker registration techniques directly to the known encoder-derived joint angles of a bipedal humanoid robot.

## Methods

We quantified the effects of OpenSim marker registration error on kinematic estimates by performing motion capture experiments on a bipedal humanoid robot with known geometry, joint definitions, gait kinematics, and joint moments, as described below.

### Robot model and description

**Fig 1:**
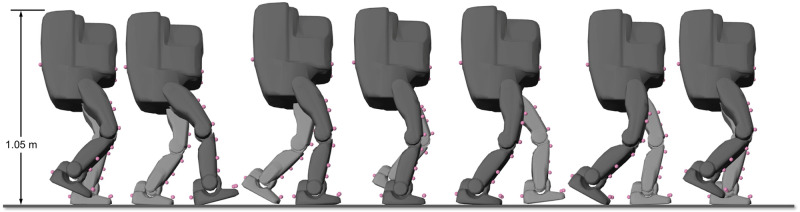
OpenSim model of a humanoid robot during gait. Model markers (pink spheres) track the measured trajectories of experimental markers (not shown) to produce a simulation of the robot’s motion. Step length is 0.33 m.

We used a torque-actuated, child-sized humanoid robot for this study (Fig 1). The robot had twelve lower-extremity degrees of freedom: three at each hip (flexion/extension, abduction/adduction, and internal/external rotation), one at each knee (flexion/extension), and two at each ankle (flexion/extension and inversion/eversion). The robot was programmed to walk at a slow speed (0.4 m/s). We used the technical specifications of the robot to build a computational model in OpenSim 4.1 with matching geometry, joint types, reference frame definitions, and inertial properties. The OpenSim model, scripts, and data underlying the findings of this study are available at https://simtk.org/projects/marker_reg, enabling others to reproduce and extend our results.

### Experimental motion capture

We affixed a set of optical markers to the robot similar to the set used in Kadaba et al. [12] and Besier et al. [26]. We used thirty-five retroreflective markers: three markers were attached to each foot, shank, and thigh; two were used to track the flexion axes of each ankle and knee; and four, two, and three markers were affixed to the pelvis, trunk, and head, respectively. The robot walked with very little arm swing; the motion of the arms was neither measured nor simulated. We used an eight-camera Vicon system (Oxford Metrics, Oxford, United Kingdom) to collect marker motion data at 200 Hz and two Bertec force plates (Bertec Corp., Columbus, Ohio) to measure ground reaction forces at 2 kHz. Marker trajectories and ground reaction force profiles were each filtered using a fourth-order low-pass Butterworth filter with a cut-off frequency of 8 Hz. We collected one static calibration trial and several walking trials. The robot’s joint encoder data were collected at 200 Hz for each lower-limb degree of freedom. Motion capture data and robot encoder data were synchronized offline using Matlab^®^ (The Mathworks, Inc., Natick, Massachusetts).

### Marker registration methods

**Fig 2:**
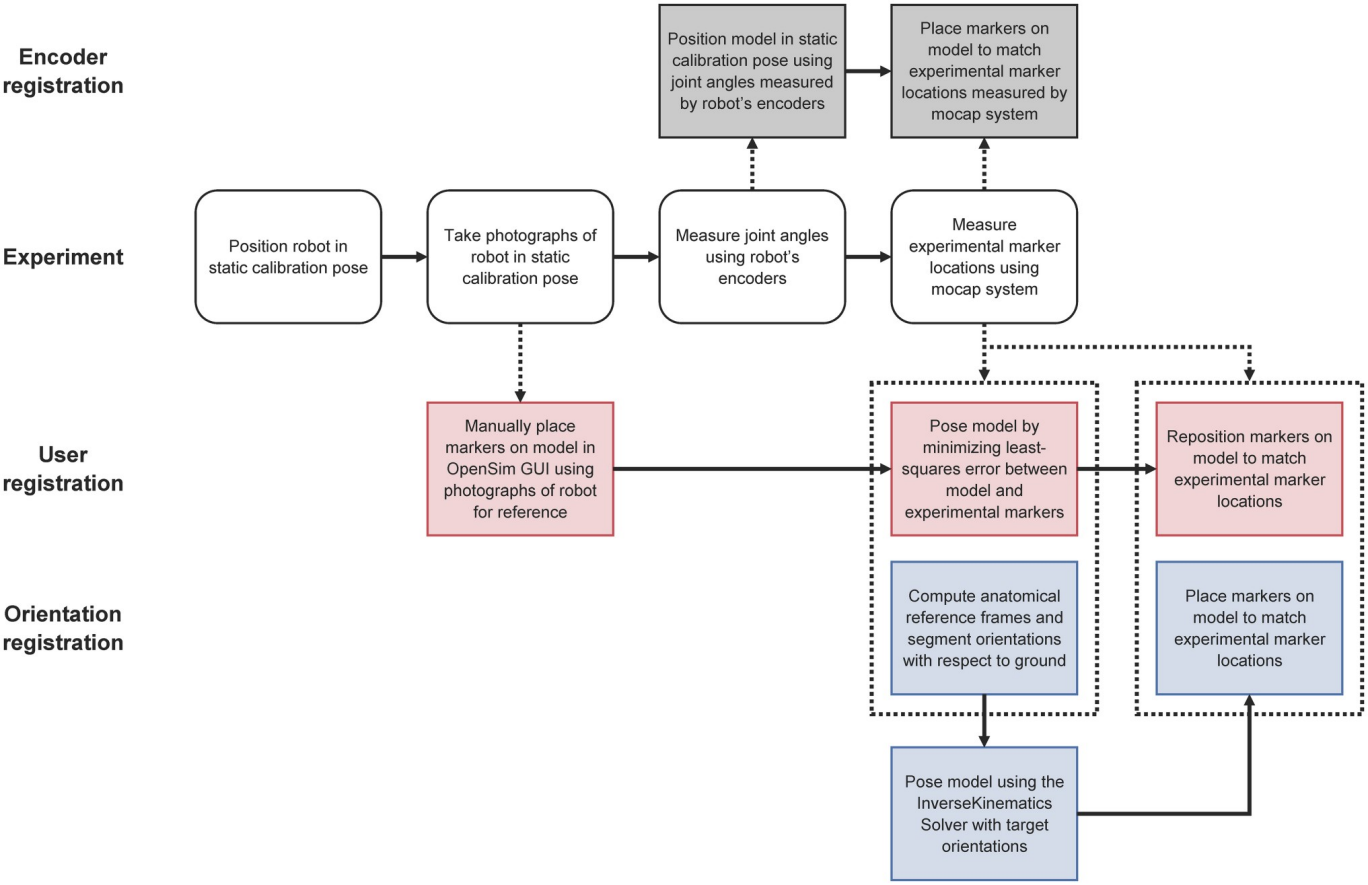
Marker registration methods examined in this study. Data collected during experiments (white boxes) were used in three registration methods: encoder registration (gray boxes), user registration (red boxes), and orientation registration (blue boxes). Solid arrows indicate the sequence in which processes occur; dotted arrows and boxes indicate the processes that use each type of experimental data.

We investigated three marker registration methods using OpenSim: one idealized method using joint encoder data from the robot and two methods that apply to human testers. These three methods are summarized in Fig 2 and described in detail below.

#### Encoder registration

In the first registration method, we positioned the OpenSim model using the joint encoder data collected from the robot during the static trial. Each model marker was then placed on the model to match the location of the corresponding experimental marker. Upon completing this procedure, the total marker error was zero relative to the static trial. We refer to this method as *encoder registration*.

#### Orientation registration

Similar to encoder registration, the model was positioned using orientation information and then model markers were automatically adjusted to match the known experimental marker locations. The orientation information was determined to match the orientations of anatomical reference frames calculated from the experimental markers (rather than measurements from the robot’s joint encoders as in the encoder registration method). We used anatomical markers at the robot’s feet, joint axes, and pelvis to define anatomical reference frames [12], and calculated segment orientations with respect to ground. Hip joint centers were determined from the robot’s technical specifications (in human subjects, hip joint centers can be determined using functional trials [26]). The InverseKinematicsSolver in OpenSim 4.1 was used to pose the OpenSim model with the best-fit anatomical segment orientations. The InverseKinematicsSolver minimizes the weighted least-squares distance between model and experimental markers [4]. Finally, markers were placed on the model to match the locations of the experimental markers. Upon completing this procedure, the total marker error was zero relative to the static trial. We refer to this method as *orientation registration*. Note that the encoder registration and orientation registration methods are algorithmic and therefore do not rely on the experience of the user.

#### User registration

Five OpenSim users, each with at least one year of OpenSim experience, manually positioned the markers on the robot model aided by photographs of the robot’s experimental static pose. We then used the OpenSim Scale Tool to complete the marker registration procedure as follows. The model was posed to approximate the robot’s configuration in the static trial by minimizing the least-squares error between model and experimental markers. (The Scale Tool performs this calculation using the InverseKinematicsSolver, described previously.) Each model marker was then repositioned to match the location of the corresponding experimental marker, rendering the total marker error zero relative to the static trial. We refer to this method as *user registration*.

### Computation and analysis

We performed constrained inverse kinematics and inverse dynamics calculations in OpenSim using experimental marker trajectories and ground reaction force profiles for one gait cycle. We repeated these calculations using each of the seven registered models: one encoder registration model, one orientation registration model, and five user registration models. The resulting kinematics were normalized to percent gait cycle. Because the stride length of the robot was insufficient to obtain complete left and right single-foot stances on two separate force plates, we computed inverse dynamics joint moments for single-leg stance only.

Accuracy of inverse kinematic joint angles and marker trajectories were determined for the three registration methods using the root-mean-square error (RMSE) between right-leg model estimates and experimentally measured right-leg encoder kinematics and marker trajectories, respectively. Due to a lack of moment-based encoder values, accuracy of joint moments was quantified for the orientation-registered and user-registered models by computing the RMSE between those moment estimates and the moment estimates from the encoder-registered model.

## Results

**Fig 3:**
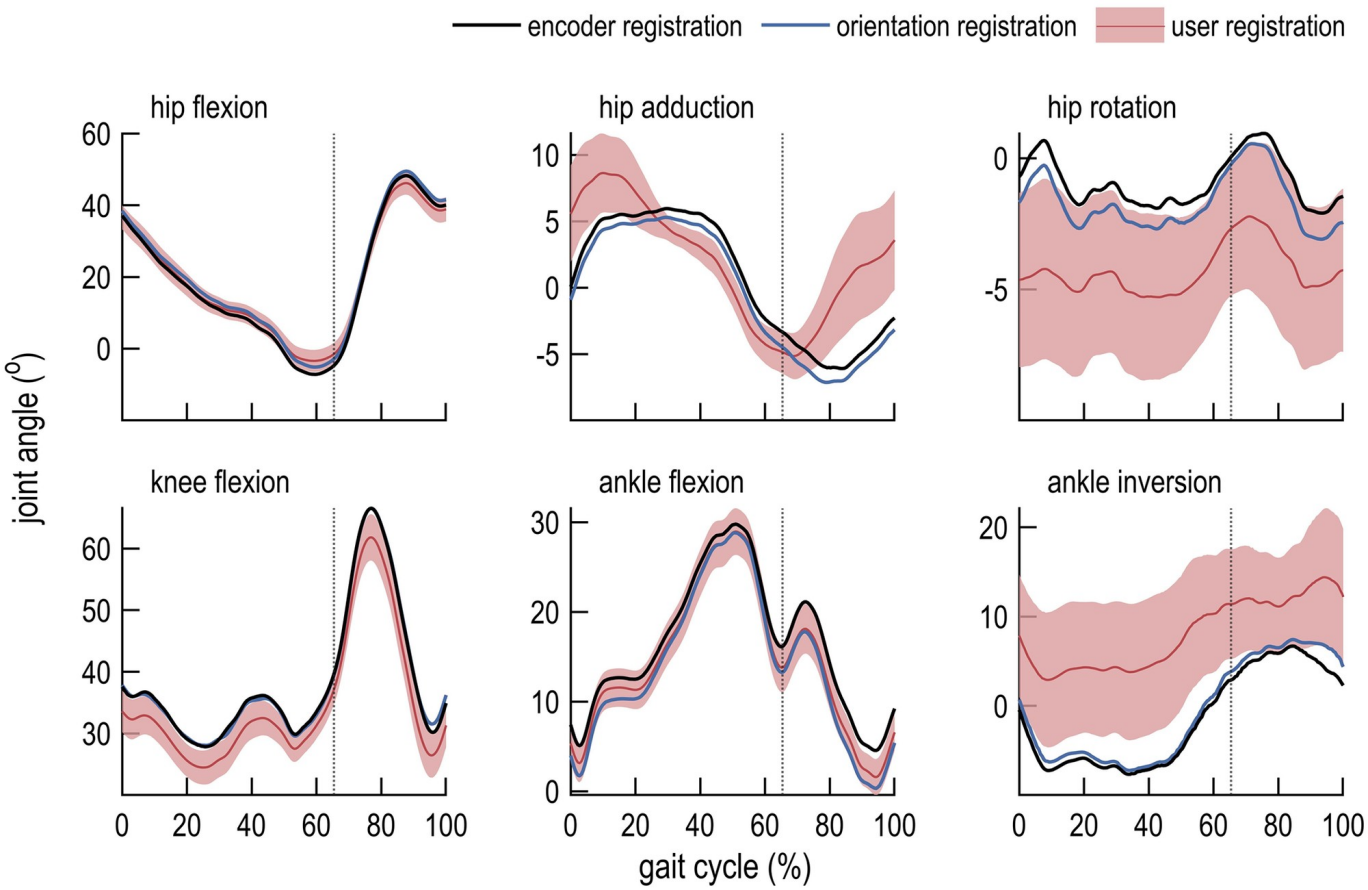
Inverse kinematics computed using three registration methods. Joint angles were computed over one gait cycle using encoder registration (black line), orientation registration (blue line), and user registration (mean, red line; standard deviation, shaded). Toe-off is indicated by a dotted vertical line.

**Fig 4:**
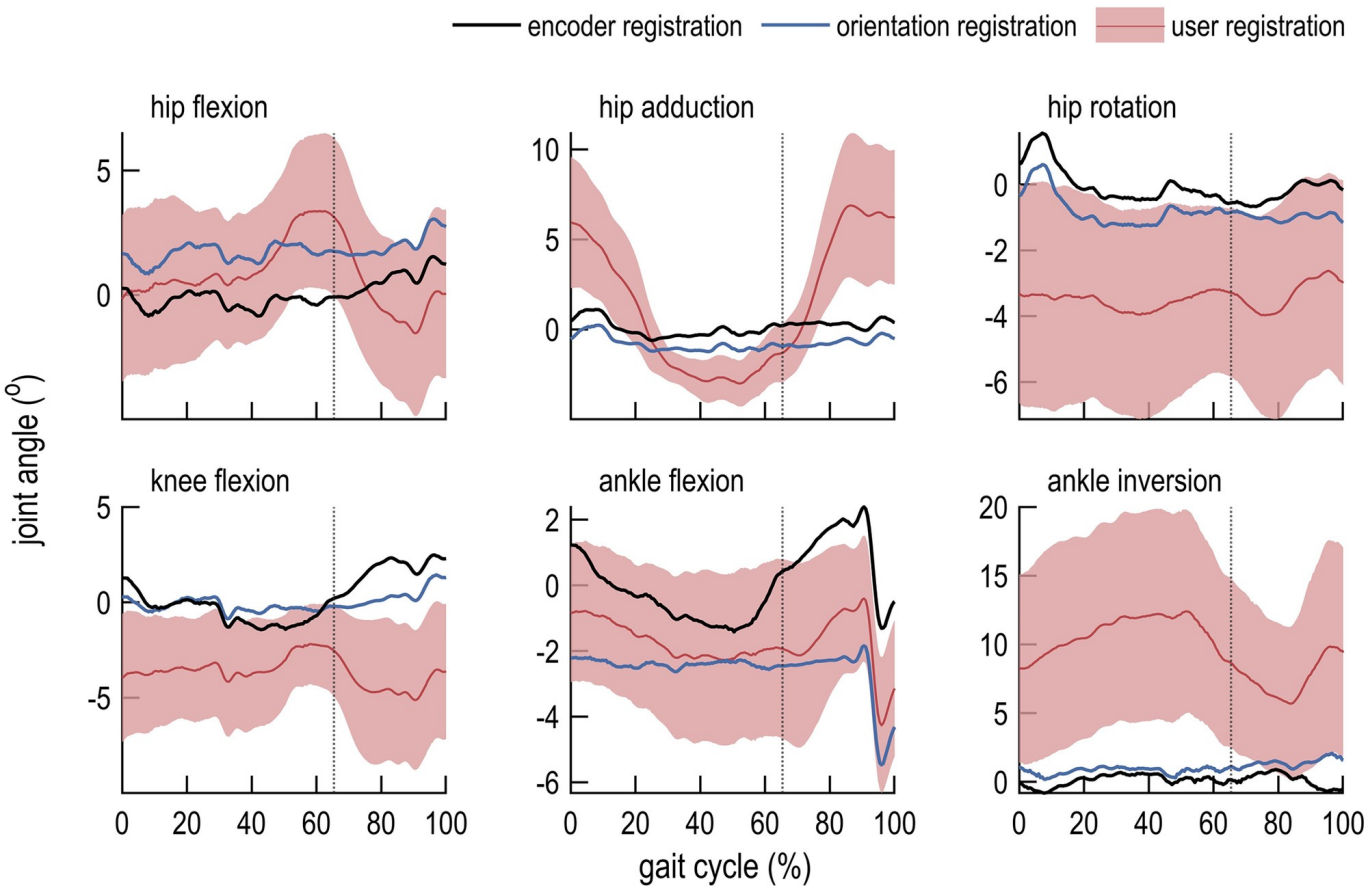
Errors in inverse kinematics calculations for each registration method. Errors in joint angles were computed relative to robot joint encoder data over one gait cycle using encoder registration (black line), orientation registration (blue line), and user registration (mean, red line; standard deviation, shaded). Toe-off is indicated by a dotted vertical line.

**Fig 5:**
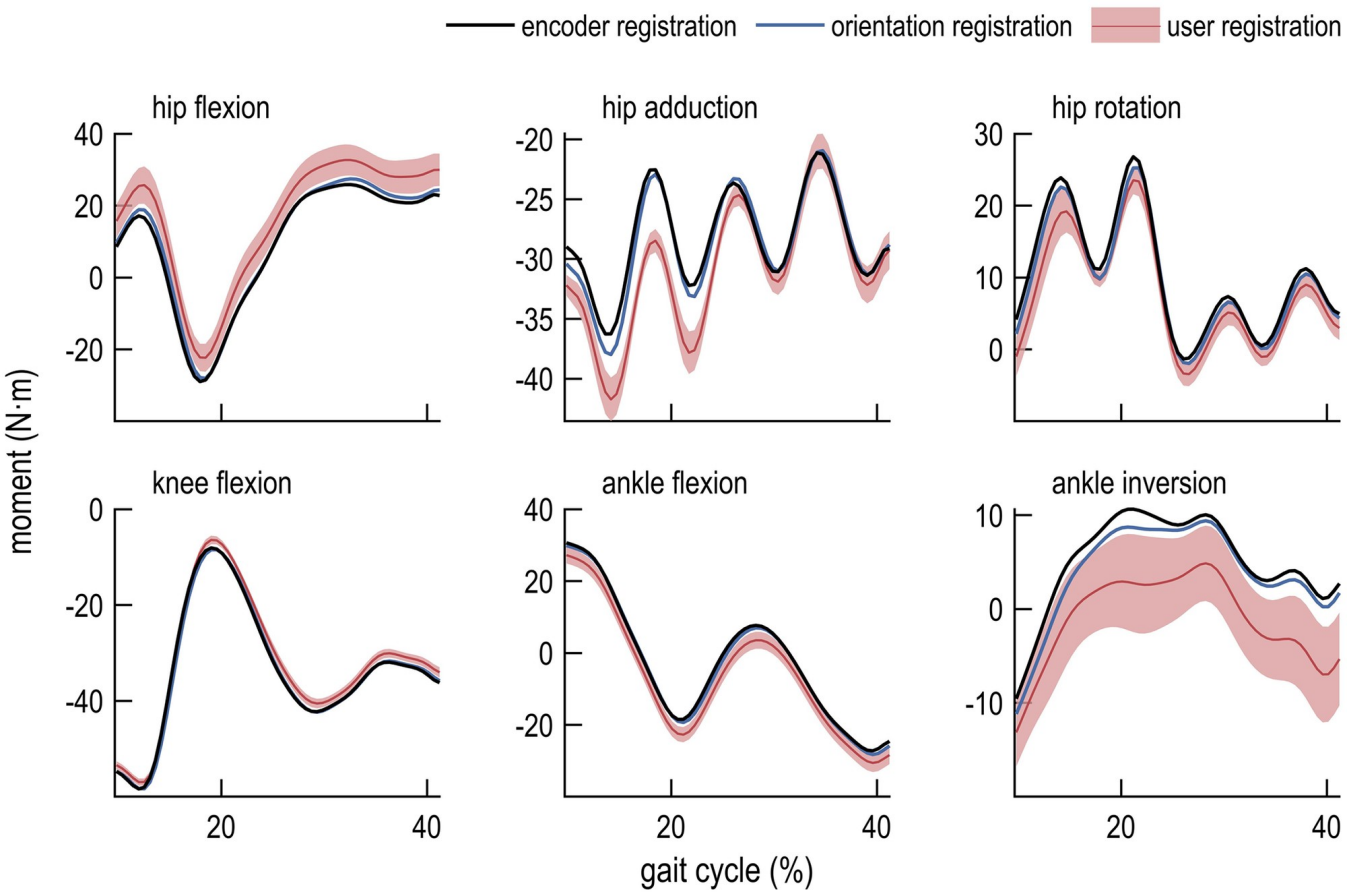
Inverse dynamics computed using three registration methods. Joint moments were computed during single-leg stance using encoder registration (black line), orientation registration (blue line), and user registration (mean, red line; standard deviation, shaded).

**Fig 6:**
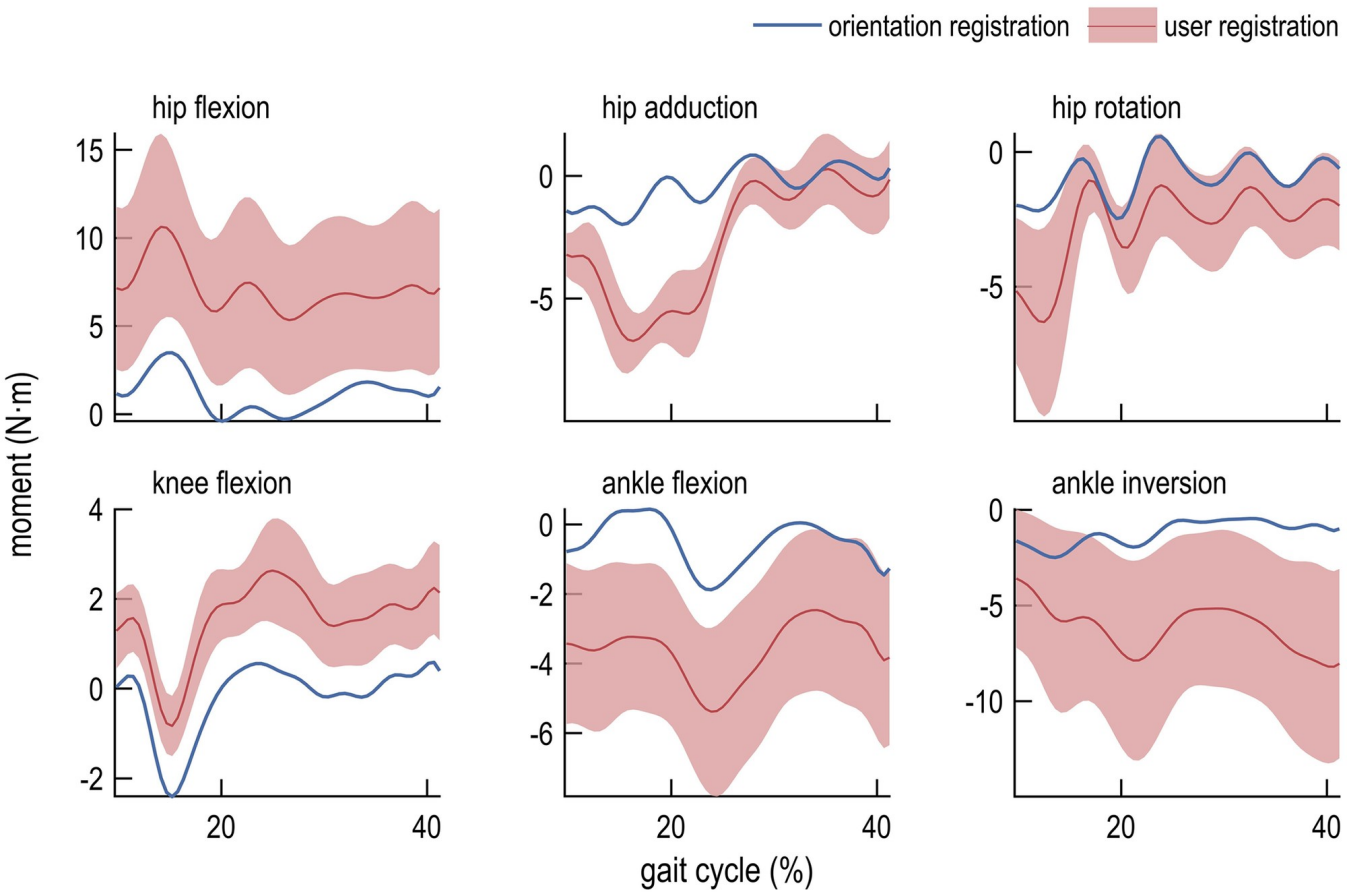
Errors in inverse dynamics calculations for orientation and user registration methods. Errors in joint moments were computed relative to the joint moments from the encoder-registered model during single-leg stance using orientation registration (blue line) and user registration (mean, red line; standard deviation, shaded).

We compared the computed joint kinematics (Fig 3), kinematic errors (Fig 4), moments (Fig 5), and moment errors (Fig 6); comparisons of RMSE are shown in Table 1. Of the registration methods examined in this study, encoder registration most accurately estimated the robot joint angles. User registration resulted in inter-user differences for all joint angles and moments. Orientation registration demonstrated lower kinematic and moment error across all degrees of freedom but one (ankle flexion) when compared to mean values obtained using user registration. On average across all lower-extremity joints, orientation registration reduced RMSE in joint angles from 3.69° to 1.21° (67%) compared to mean values obtained with user registration, and reduced RMSE in joint moments from 3.37 N⋅m to 1.11 N⋅m (67%). Marker RMSE was smallest for encoder- and orientation-registered models. Notably, unlike the user registration method, the encoder and orientation registration methods do not exhibit inter-user variability since they do not rely on user input.

**Table 1:** Joint angle and joint moment errors for each marker registration method examined in this study. Joint angle accuracy (“Angle”) is computed as the RMSE between inverse kinematics calculations and robot encoder data; joint moment accuracy (“Moment”) is computed for the orientation- and user-registered models as the RMSE between inverse dynamics calculations and the estimated moments from the encoder-registered model. Mean and standard deviations across the five OpenSim users are shown for the user-registered models. Mean and standard deviation of marker RMSE are computed for each registration method over all time points.

	Encoder registration		Orientation registration		User registration	
	Angle (deg)	Moment (N⋅m)	Angle (deg)	Moment (N⋅m)	Angle (deg)	Moment (N⋅m)
Hip flexion	0.46	—	1.76	1.54	2.12 ± 1.30	6.36 ± 3.69
Hip adduction	0.42	—	0.85	0.91	3.54 ± 1.68	2.73 ± 1.30
Hip rotation	0.55	—	0.88	1.21	2.71 ± 2.40	2.34 ± 1.50
Knee flexion	1.16	—	0.33	0.80	3.05 ± 2.37	1.48 ± 0.65
Ankle flexion	1.07	—	2.38	0.85	1.99 ± 1.06	2.82 ± 2.04
Ankle inversion	0.46	—	1.04	1.35	8.75 ± 5.05	4.46 ± 3.59
Marker error (mm)	2.3 ± 1.2		2.3 ± 1.2		12.9 ± 7.0	

## Discussion

In this study, we isolated marker registration error from other sources of kinematic error, such as improper model scaling, soft tissue artifacts, and joint center estimation. All registration methods were evaluated using the same robot model. We showed that marker registration can have a substantial effect on joint angles and moments computed using constrained inverse kinematics in OpenSim. However, we demonstrated that the RMSE between OpenSim model and experimental markers was consistent and small (less than 13 mm) regardless of the marker registration method. These results indicate that, even if mean marker error is small, joint angles obtained from constrained inverse kinematics may still be inaccurate. Orientation registration was shown to be more accurate than user registration when performing constrained inverse kinematics with an OpenSim model of a bipedal robot.

The joint angle differences between models appeared as offsets in otherwise similar trajectories (Fig 3). The least variability in joint angles was observed in the sagittal plane. Similar results were reported by Lathrop et al. [27] when evaluating kinematic estimates between OpenSim and the point cluster technique. Lathrop et al. suggested that the observed differences may be due to the cumulative effects of differences in model geometry, model marker positions, and anatomical reference frame definitions. Our study isolated model marker registration error and demonstrated its effects on calculations of joint angles and moments. Further work is necessary to understand how geometric differences between the model and subject affect computed joint angles and moments.

The strength of this robot-based study is that it isolated the effects of marker registration without the confounding factors of soft-tissue movement and errors in model segment and joint definitions that are prevalent in human movement experiments. The main limitation of this study, however, is that we cannot directly translate these findings to human experiments. While we expect interactions between modeling errors and marker registration errors to lead to even greater inaccuracies in the calculation of joint angles and moments, how these errors are combined (e.g., additive or multiplicative) remains unknown. It is also possible, for example, that modeling errors and soft-tissue artifacts dominate the errors due to marker registration and that the latter are relatively unimportant, particularly during movements with high accelerations. These speculations are difficult to address without first quantifying the contributions of each source of error. This is the first study to isolate and quantify the contribution of marker registration error. Future studies should quantify the effect of other sources of error, such as incorrect scaling, joint center locations, and measurement noise, and provide recommendations for experimental methods to mitigate the largest sources of error.

Using a robot for this analysis provided unique insight into the errors introduced by marker registration for studies involving human subjects, where differences between the biomechanical model and experimental subject may be greater and anatomical landmarks may be more difficult to identify. It is important to be aware of marker registration error and its potential effect on the joint angles computed using constrained inverse kinematics. An inverse kinematics method will compute a single trajectory for each joint angle over time, but the uncertainty associated with inverse kinematics calculations must not be ignored when interpreting the results of a study. To reduce OpenSim marker registration error, we recommend computing anatomical reference frames from the skin-mounted markers to determine the pose used to register markers on the model. Our findings show that the accuracy of model kinematics can be improved by using anatomical reference frames to pose models and register markers before computing joint angles using constrained inverse kinematics.
