# Correction: A marker registration method to improve joint angles computed by constrained inverse kinematics

**DOI:** 10.1371/journal.pone.0254509

**Published:** 2021-07-07

**Authors:** James J. Dunne, Thomas K. Uchida, Thor F. Besier, Scott L. Delp, Ajay Seth

The second author, Thomas K. Uchida, is incorrectly noted as the corresponding author. The correct corresponding author is Ajay Seth. Dr. Seth’s email address is: a.seth@tudelft.nl.
